# Infantile Spinal Paralysis, (Anterior Poliomyelitis)

**Published:** 1916-08

**Authors:** Theodore Toepel

**Affiliations:** 1625-26 Candler Bldg., Atlanta, Ga.


### Journal-Record of Medicine

Successor to Atlanta Medical and Surgical Journal, Established 1855
and Southern Medical Record, Established 1870
**OWNED BY THE ATLANTA MEDICAL JOURNAL COMPANY**
PUBLISHED MONTHLY

*Official Organ Fulton County Medical Society, State Examing Board, Atlanta, Birmingham and Atlantic Railroad Surgeon's Association, Chattahoochee Valley Medical and Surgical Association, Etc.*

A. W. STIRLING, M. D., C. M., D. P. H.; J. S. HURT, B. Ph., M.D.
GEO. M. NILES, M. D., W. J. LOVE, M. D., (Ala.); Associate Editors
E. W. ALLEN, Business Manager

**COLLABORATORS**

W. F. WESTMORELAND, M. D., General Surgery
F. W. McRAE, M. D., Abdominal Surgery
ALLEN H. BUNCE, Medical Societies
E. B. BLOCK, M. D., Diseases of the Nervous System
MICHAEL HOKE, M. D., Orthopedic Surgery
CYRUS W. STRICKLER, M. D., Legal Medicine and Medical Legislation
E. C. DAVIS, A. B., M. D., Obstetrics
E. G. JONES, A. B., M. D., Gynecology
ALLEN H. BUNCE, M. D., Pathology and Bacteriology
R. T. DORSEY, Jr., B. S., M. D., Medicine
L. M. GAINES, A. B., M. D., Internal Medicine
GEO. C. MIZELL, M. D., Diseases of the Stomach and Intestines
L. B. CLARKE, M. D., Pediatrics
EDGAR PAULLIN, M. D., Opsonic Medicine
THEODORE TOEPEL, M. D., Mechano Therapy
R. R. DALY, M. D., Diseases of the Eye, Ear, Nose and Throat
E. G. BALLENGER, M. D., Diseases of the Genito-Urinary Organs

VOL. LXIII. ATLANTA, GA., AUGUST, 1916. NO. 5

### INFANTILE SPINAL PARALYSIS.

(Anterior Poliomyelitis)

THEODORE TOEPEL, M. D.

### TREATMENT.

1. Restoration of Function.
   (a) Massage and electricity.
   (b) Gymnastic and voluntary muscular exercises.
2. Prevention of Deformity.
   (a) Splints
   (b) Mechanical appliances and apparatus.

3. Operative Treatment.

1. Restoration of Function:—In the febrile initial stage every discoverable cause should be investigated and removed. Primarily the alimentary canal must be cleansed to relieve auto infection. Dental, genital, rectal, gastric and any source of reflex irritation should be corrected.

During the acute stage (stage of onset) the body requires the physiologic rest in the prone position. During this stage joints will not stiffen, hopeless muscular atrophy will not occur, and by this proceeding the damaged cord will have the best chance to repair.

The second stage, or phase of convalescence begins with the disappearance of the tenderness at the end of two or three months and lasts for two years or more.

Restoration of function with the avoidance of fatigue must now be pursued with proper discrimination of individual conditions.

(a) Massage is of value because it empties the veins and lymphatics and thus promotes the flow of blood to the limb. It does not promote the passage of nervous impulses from brain to muscle, and its action must be considered purely local. Given too a long a time it is detrimental and retards progress. Dry heat from an electric pad or other means while the patient is in bed will give much comfort.

Electricity, properly used will be of much benefit. Faradism causes a mild muscular contraction and is a useful form of gentle exercise.

(b) Gymnastics and voluntary muscular exercises are especially to be encouraged; they are of the greatest value at this stage. Voluntary muscular exercise attempts to drive an impulse from brain to muscle to enable it if possible to open up new paths around affected centers in the cord. The connection between these centers with each other and between the centers and the muscles is most extensive, and the facts given as to the predominence of partial paralysis show that, as a rule, the entire nervous control of a given muscle is not wiped out as a whole, but only in part. On this basis rests the claim of re-education of muscles, which is one of the most powerful factors in determining ultimate muscular function. Dr. R. W. Lovett of Boston, who made a study of the Vermont epidemic collected the following data by means of muscle tests of the efficacy muscle training.

The chance of improvement in affected but not totally paralyzed muscles under expert treatment by muscle training was about 6 to 1, under supervised home exercises 3.5 to 1, under home exercises without supervision 2.8 to 1, while untreated affected muscles in these patients showed an improvement ratio of 1.9 to 1.

In young children where the helpful element of play must be secured to induce voluntary muscular movements in the limbs, appliances, such as a bicycle run by a motor, a spring swing, a baby-tender or a wheeled crutch, a spring board, a rope and board swing, a miniature merry-go-round for outdoor use, etc., are useful. In conclusion of this part of the treatment let me say that fatigue and over treatment by massage and voluntary exercises are detrimental factors of the highest importance too little attended to. The advice often given to use affected limbs as much as possible is the worst advice that can be given. It is difficult to under use such muscles, but fatally easy to injure them by over-use.

2. Prevention of Deformities.

(a) Splints:—Measures to prevent the to-be-expected deformities should begin while the patient is in bed. The common occurrence of foot-drop requires that the weight of the bed clothing and the influence of gravity shall not stretch the weakened anterior tibial muscles at the ankle. A bed hoop or cradle and a light felt, leather or metal right angled splint will maintain the position of the foot and permit removal for massage and voluntary exercise. Two conditions very frequently overlooked which lead to serious results are weakening or paralysis of the abdominal muscles and scoliosis. When these occur the use of cloth corset or plaster jacket is important from the time that the first stage is over.

(b) Mechanical Appliances and Apparatus.

Mechanical treatment is most valuable as preventive of deformity both before and after operative relief, but it is cruel to attempt for weeks or months to stretch a strong, contracted muscle and tendon by braces, when a simple tenotomy will easily and painlessly remedy the distortion in a few minutes and will improve, not lessen, contractile power. Objection is often made to braces that they produce atrophy of the limb, the fact being overlooked that the atrophy is a constant result of the disease itself.

No definite outlines can be formulated for the construction of apparatus, except that it should be individually and mechanically planned by the surgeon after a careful inspection of the nude child, after which he can decide as to the muscles that need assistance, the ones that are liable to contract, and the distortions of bones, ligaments and joints that will probably be produced by faulty weight-bearing. The chief advantage of apparatus is in post operative support.

While manipulative and mechanical treatment is of great importance in the prevention of deformities, yet unfortunately the orthopedic surgeon has most frequently to deal with the neglected and extreme distortions produced by weight-bearing upon hip, knee and ankle. The results of skillful operative measures are so demonstrable and effective that they should never be denied to any sufferers who have reasonably strong limbs. It is inhuman to condemn any person to a hopeless existence when a few moments of surgical help and a few months of patient, after-treatment. will readily result in a self-supporting and mentally elevated individual.

The object of surgical treatment is to bring feet, knees and hips into such lines of weight-bearing that either with or without mechanical support walking will be possible. In all severe cases of deformity surgical treatment should precede mechanical. Success depends upon the judgment and operative skill of the surgeon, the subsequent care, and the protection and education of the muscles.

3. Operative Treatment.

(a) Tenotomies and fasciotomies; (2) myotomy; (3) forcible correction under ether, manual and instrumental; (4) open incision; (5) tarsectomy; (6) excision; (7) osteotomy: (8) arthodesis; (9) bone lengthening; (10) tendon shortening; (11) tendon transplantation; (12) nerve anastomosis, etc.

Summary of Treatment.

1. Rest during stage of tenderness.

2. Massage and electricity in suitable cases.

3. Manipulation, stretching and friction, with gymnastic exercises.

4. Prevention of foot-drop while in bed.

5. Apparatus to prevent deformity as soon as child walks, to support the weight of the body and develop weakened muscles. The natural atrophy of the disease, not the brace, will result in shrinkage of the limb. Deformity is usually the result of neglect of precautionary measures.

6. In contracted and deformed joints, surgical interference to bring limbs into straight line and walking position with the body is far more speedy and humane than instrumental stretching.

7. Artificial mechanical support by braces after operation is usually required, with or without canes or crutches, with systematic and gymnastic muscular development for a year or more.

8. Any form of locomotion is better than helpless dependance. No patient possessing sufficient arm power to steady himself upon crutches should be considered hopeless. Carefully planned and executed surgical, mechanical and muscular developmental methods will result in utilizing the legs and arms for some sort of locomotion if perseveringly followed.

1625-26 Candler Bldg., Atlanta, Ga.

